# Pioneering hospital-at-home in Taiwan: early clinical outcomes from the first cohort of nursing home older adults

**DOI:** 10.3389/frhs.2025.1696104

**Published:** 2025-12-12

**Authors:** Shang-Lin Chou, Shih-Tien Chen, Jen-Pin Chuang

**Affiliations:** 1 Departrment of Emergency Medicine, Chiayi Hospital, Ministry of Health and Welfare, Chiayi, Taiwan; 2 Department of Recreational Sport and Health Promotion, National Pingtung University of Science and Technology, Pingtung, Taiwan; 3 Departrment of Internal Medicine, Chiayi Hospital, Ministry of Health and Welfare, Chiayi, Taiwan; 4 Department of Surgery, Chiayi Hospital, Ministry of Health and Welfare, Chiayi, Taiwan; 5 Department of Surgery, Faculty of Medicine, College of Medicine, National Cheng Kung University, Tainan, Taiwan

**Keywords:** hospital-at-Home (HaH), Taiwan older adults, nursing home, pneumonia, urinary tract infections (UTIs), soft tissue infections (STIs)

## Abstract

**Objective:**

This study provides the first empirical evaluation of Taiwan's Hospital-at-Home (HaH) pilot program, launched in 2024 under the National Health Insurance system. The aim was to examine the clinical effectiveness, safety, and economic feasibility of HaH in managing acute infections, including pneumonia, urinary tract infections (UTIs), and soft tissue infections (STIs), among older adults living in long-term care facilities.

**Methods:**

A prospective, matched-controlled study was conducted from July 2024 to June 2025 across seven nursing homes. Sixty residents aged 65 years or older who received HaH care were matched in a 1:2 ratio with 120 hospitalized patients by age, sex, and diagnosis. HaH services were delivered by a single interdisciplinary team. Primary outcomes included care duration, medical costs (USD), emergency department (ED) revisits, readmissions, and mortality. Secondary outcomes were complication rates. Statistical analyses used Chi-square tests, t-tests, and Mann–Whitney U tests, with odds ratios and 95% confidence intervals reported. A *p*-value < 0.05 was considered significant.

**Results:**

HaH patients had significantly shorter care episodes compared with hospitalized patients (6.6 ± 1.5 vs. 11.8 ± 6.0 days, *p* < 0.001) and lower medical costs across all diagnoses. For STIs, costs were reduced by 65.1% (USD 979 vs. 2,805, *p* < 0.001), while UTIs and pneumonia showed savings of 46.0% and 45.5%, respectively. Overall clinical outcomes, including ED revisits, readmissions, and mortality, were similar between groups. In the STI subgroup, HaH patients had a significantly lower 14-day ED revisit rate (7.4% vs. 27.8%, odds ratio 0.21, 95% confidence interval 0.04–0.99, *p* = 0.04). HaH patients also experienced fewer hospital-acquired complications, particularly gastrointestinal and neurological events.

**Conclusion:**

The findings demonstrate that HaH is a safe, effective, and cost-efficient alternative to hospitalization for acute infections in institutionalized older adults. By reducing care duration and costs without compromising clinical outcomes, HaH offers a patient-centered model that can ease healthcare system pressures in rapidly aging societies. These results support further expansion of HaH in Taiwan and encourage additional longitudinal studies to confirm long-term benefits and broader health system impacts.

## Introduction

Since the implementation of Taiwan's National Health Insurance (NHI) system in 1995, the country has been praised for providing affordable and widely accessible healthcare, achieving over 99% population coverage and high patient satisfaction (1, 2). However, the nation continues to face challenges in achieving optimal health outcomes, particularly when compared to other OECD countries (3, 4), and especially in the context of its rapidly aging population. As of recent estimates, Taiwan has over 4.2 million people aged 65 and older—about 17.6% of its total population—and this is expected to surpass 20% by 2025, marking Taiwan as a “super-aged” society (5). Among adults aged 65 and older, approximately 1.9% reside in institutional long-term care facilities such as nursing homes or senior care centers, accounting for over 82,000 individuals (6). Hospital care remains the largest healthcare expenditure (7, 8) and poses heightened risks for patients, including hospital-acquired complications such as infections, falls, functional decline, and cognitive deterioration (9, 10).

Hospital at Home (HaH) has emerged as a successful model in several developed countries (11). These programs have demonstrated comparable or better clinical outcomes than traditional hospital care, with benefits such as reduced hospital readmissions, lower healthcare costs, and improved patient satisfaction. For instance, a systematic review by Shepperd et al. (2021) found that HaH reduced mortality and readmission rates in older patients compared to inpatient care (12). In the U.S., Levine et al. (13) reported that an acute care HaH model decreased the cost of care by 38% while maintaining quality and safety (13). In contrast, institutional care settings are often associated with high rates of polypharmacy, hospital-acquired complications, and frequent overuse of emergency services (14–17), making HaH a potentially safer and more cost-effective alternative.

In response to the frequent hospital admissions among older residents, particularly those with dementia, frailty, or declining functional capacity, the Taiwan's National Health Insurance Administration(NHIA) launched the HaH pilot program in July 2024 (18). By enabling timely and targeted treatments within familiar environments, HaH has the potential to reduce unnecessary hospitalizations, improve patient comfort, and enhance continuity of care, especially for the vulnerable older people in nursing homes. This initiative aims to transition acute care services from hospitals to patients' homes or care facilities, focusing on three common geriatric infections: pneumonia, urinary tract infections (UTIs), and soft tissue infections (STIs), which are leading causes of hospitalization in this demographic (19). According to data from Taiwan's NHIA in Sep 2024 (20), the HaH pilot program has involved 160 teams, 692 healthcare institutions, and 3,571 medical professionals. The program provides in-home medical care for patients with pneumonia, urinary tract infections (UTIs), and skin and soft tissue infections (STIs). As of August 2024, 71 institutions had enrolled patients, with a total of 255 cases under care. Regional hospitals and clinics accounted for 32 percent and 28 percent of these cases, respectively, together comprising 60 percent of the total. Despite these efforts, the effectiveness of implementing home-based medical care in Taiwan remains unclear.

This study aims to be the first to evaluate the effectiveness of the HaH model in Taiwan, providing empirical evidence on clinical outcomes, care quality, and feasibility in long-term care institutions. The pilot program represents a critical shift toward more sustainable and patient-centered healthcare delivery as Taiwan prepares to meet the growing demands of an aging society.

## Materials and methods

### Study design and setting

This was a prospective, matched-controlled study conducted between July 2024 and Jun 2025 across seven nursing homes in Taiwan. The study evaluated the clinical outcomes of the HaH initiative under Taiwan's NHIA pilot program for acute home care. The HaH services were provided by an interdisciplinary care team composed of physicians, nurses, respiratory therapists, and pharmacists, all affiliated with Chia-yi Hospital, Ministry of Health and Welfare, an NHI-contracted healthcare institution. Care services included daily bedside visits by nursing staff, physician home visits or telemedicine monitoring, bedside diagnostic testing, medication reconciliation, remote vital signs monitoring via IoT devices, 24-hour emergency consultation services, and coordination with long-term care systems. A matched control group was identified from the same hospital's inpatient records during the same period, with admission dates for both groups within three months, for outcome comparisons.

### Participants

Participants in the HaH group were nursing home residents aged 65 years or older who had been assessed by attending physicians as presenting with acute medical conditions typically requiring hospitalization. Eligible diagnoses included pneumonia (ICD-10 codes: J12–J18, J20–J22, J69.0), urinary tract infections (N10, N30.0, N30.3, N30.8, N30.9, N34, N39.0), and skin or soft tissue infections (L03.0–L03.9). In addition to meeting diagnostic criteria, participants had to fulfill at least one of the following eligibility requirements: enrollment in an existing home care or respiratory dependency care program, residency in a long-term care facility participating in the national “Reduction of Institutional Transfers” initiative, or a recent emergency department visit with a Barthel Index score below 60 or significant mobility impairment that would hinder safe or practical access to hospital-based care. A total of 60 patients met these inclusion criteria and were enrolled in the HaH group.

To create a comparable control group, 120 hospitalized patients were matched 1:2 to HaH participants based on age, sex, primary diagnosis, and timing of hospitalization. All control patients had received standard inpatient care during the same study period. Matching was performed to minimize potential confounding effects and to enhance comparability between the groups.

### Variables

Clinical and demographic data were obtained through standardized case report forms, electronic health records, and structured patient and family surveys. These data sources provided comprehensive information on baseline characteristics, care processes, and post-discharge outcomes. Data were derived from standardized institutional documentation systems used across both HaH and hospital care, ensuring consistent definitions and data formats. The primary outcomes measured were the length of the care episode (in days), medical cost (in USD), rates of emergency department (ED) revisits within 3 and 14 days, hospital readmissions within 3 and 14 days, and all-cause mortality within 7 and 30 days following the initial episode of care. Because NHIA reimbursement bundles aggregate medical costs across multiple fee categories, total reimbursed cost was used as the standardized cost metric. All-cause revisits and readmissions were used to maintain objectivity and avoid retrospective misclassification. Secondary outcomes included the incidence of complications commonly observed in older or critically ill patients, such as hospital-acquired infections (e.g., pneumonia, UTIs, and surgical site infections), cardiovascular and respiratory complications (including deep vein thrombosis and pulmonary embolism), gastrointestinal problems (such as gastrointestinal bleeding or constipation), neurological issues (especially delirium), and others issues including metabolic imbalances, pressure ulcers, falls, and adverse drug reactions. All procedures and data handling complied with the ethical standards required for participation in the NHI pilot program, and all identifiable patient data were de-identified to ensure confidentiality.

### Statistical analysis

Baseline characteristics between the HaH group and the hospital-based control group were compared using Chi-square tests for categorical variables and independent samples *t*-tests for continuous variables. The length of care episodes was analyzed using the Mann–Whitney U test due to non-normal distribution. Clinical outcomes including emergency department (ED) revisits within 3 and 14 days, hospital readmissions within 3 and 14 days, and all-cause mortality within 7 and 30 days following the initial episode of care were evaluated using Chi-square tests. Results were reported as odds ratios (OR) with 95% confidence intervals (CI). All statistical analyses were conducted using SPSS version 26.0, with a two-sided *p*-value of <0.05 considered statistically significant.

## Results

### Patient characteristics

A total of 180 participants were included in this study, comprising 60 patients in the HaH group and 120 patients in the hospital-based control group. The two groups were well-matched in terms of age, sex, functional status, and primary diagnosis, as summarized in Table 1. The mean age of participants was 77 years, with no statistically significant difference between the HaH and control groups (77.0 ± 6.3 vs. 76.7 ± 5.8 years, *p* = 0.85). The proportion of male participants was identical in both groups (56.7%), and the distribution of primary diagnoses was also equivalent, with STIs being the most common (45.0%), followed by UTIs at 43.3%, and pneumonia at 11.7% in both groups (*p* = 1.00 for all). Functional status, assessed using the Barthel Index, indicated a high level of dependency in both groups, though the HaH group had a slightly higher mean Barthel score compared to the control group (3.9 vs. 2.3), which did not reach statistical significance (*p* = 0.17). Most patients scored in the lowest functional category (0–20), representing 95.0% in the HaH group and 97.8% in the control group (*p* = 0.11), with very few scoring above 20. These results suggest that the HaH and hospital-based groups were comparable in demographics and baseline clinical characteristics, including age, gender, diagnosis distribution, and functional dependency levels, supporting the validity of subsequent outcome comparisons between the two cohorts.

**Table 1:** Baseline characteristics of the study population.

Characteristic	Hospital-at-home (*n* = 60)	In Hospital control (*n* = 120)	*p*-value
Age, mean ± SD (years)	77.0 ± 6.3	76.7 ± 5.8	0.85
Male, *n* (%)	34 (56.7%)	68 (56.7%)	1.00
Barthel Index			
mean (range)	3.9 (0–35)	2.3 (0–35)	0.17
0–20	57 (95.0%)	176 (97.8%)	0.11
>20	3 (5.0%)	4 (2.2%)	
Primary diagnosis *n* (%)			
Pneumonia	7 (11.7%)	14 (11.7%)	1.00
UTIs	26 (43.3%)	52 (43.3%)	1.00
STIs	27 (45.0%)	54 (45.0%)	1.00

### The length and cost of the care episode

**Figure 1:**
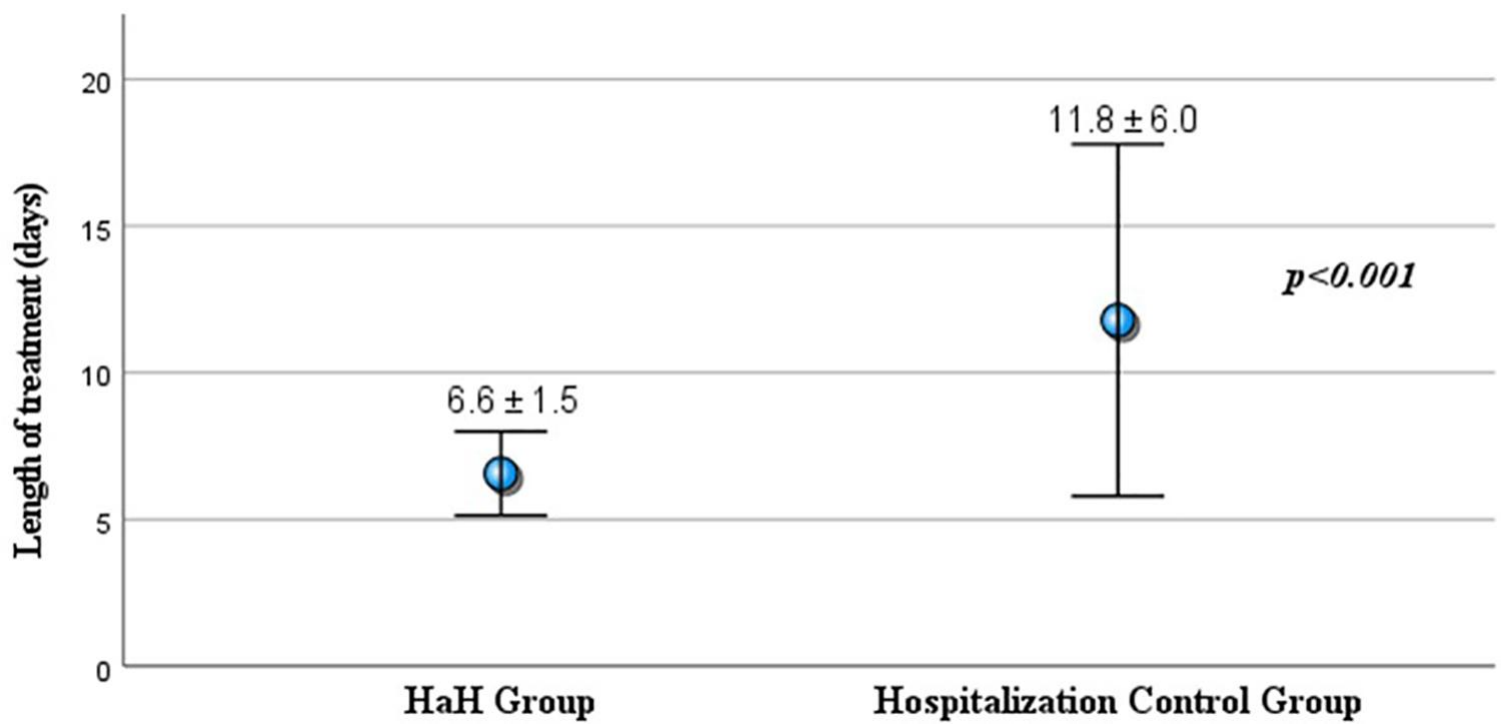
Length of treatment (mean ± SD in days) for HaH group (*n* = 60) vs. In-Hospital Control Group (*n* = 120).

**Figure 2:**
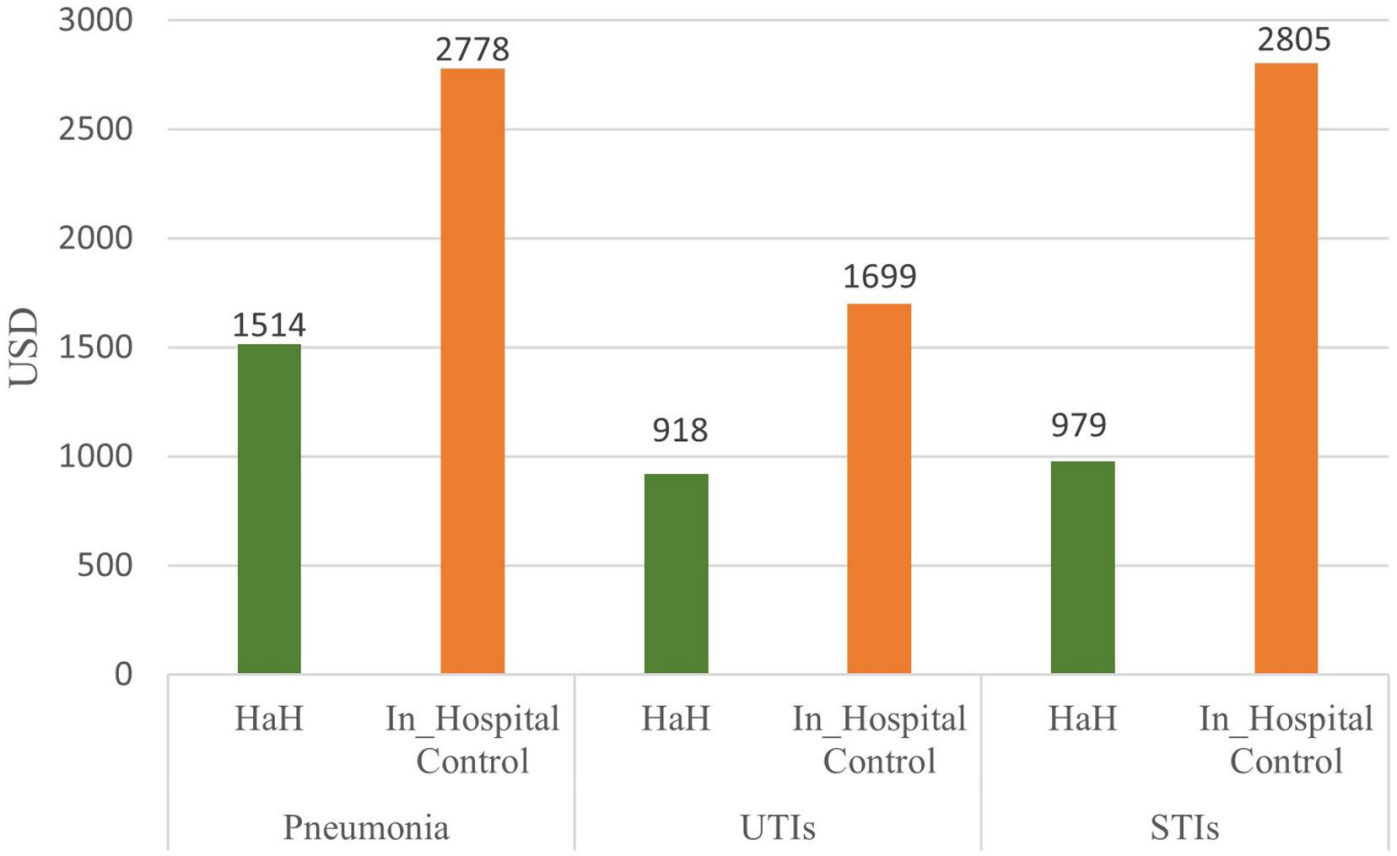
Comparison of mean healthcare costs (USD) between HaH and in-hospital control groups across three infection types.

As shown in Figure 1, the length of the care episode was significantly shorter in the HaH group, with a mean duration of 6.6 ± 1.5 days, compared to 11.8 ± 6.0 days in the in-hospital control group. This difference was statistically significant (*p* < 0.001). Subgroup analysis by primary diagnosis in Table 2 demonstrated consistently shorter care durations in the HaH group compared to the in-hospital control group. Among patients with pneumonia, the HaH group had a mean care episode of 8.4 ± 1.6 days vs. 15.1 ± 7.4 days in the control group (*p* = 0.005). For UTIs, the HaH group averaged 6.3 ± 1.2 days, significantly less than the 10.3 ± 4.2 days in-hospital (*p* < 0.001). Similarly, patients with STIs had shorter stays in the HaH group (6.3 ± 1.3 days) compared to the in-hospital group (12.4 ± 6.6 days, *p* < 0.001). In the analysis of mean healthcare costs, patients treated under the HaH model incurred significantly lower expenses across all diagnostic categories compared to those receiving traditional in-hospital care (Figure 2). For pneumonia cases, the average cost for HaH patients was USD 1,514, compared to USD 2,778 for hospitalized patients, representing a 45.5% cost reduction (*p* = 0.005). In urinary tract infections (UTIs), HaH patients averaged USD 918, while hospital care costs reached USD 1,699, reflecting a 46.0% savings (*p* < 0.001). The most substantial difference was observed in soft tissue infections (STIs), where the average cost for HaH was USD 979, in contrast to USD 2,805 in the hospital group—a 65.1% reduction (*p* < 0.001).

**Table 2:** Length of treatment for HaH group (*n* = 60) vs. In-Hospital Control Group (*n* = 120) in different dianosises.

Diagnosis	Group	Mean ± SD (days)	95% CI (days)	*p*-value
Pneumonia (*n* = 21)	HaH	8.4 ± 1.6	7.2–9.7	0.005^a^
	In_Hospital Control	15.1 ± 7.4	11.2–19.3	
UTIs (*n* = 78)	HaH	6.3 ± 1.2	5.8–6.8	<0.001^a^
	In_Hospital Control	10.3 ± 4.2	9.1–11.4	
STIs (*n* = 81)	HaH	6.3 ± 1.3	5.9–6.9	<0.001^a^
	In_Hospital Control	12.4 ± 6.6	10.8–14.2	

a *p* < 0.05.

### Clinical outcomes

The clinical outcomes comparing HaH and traditional in-hospital care for patients with pneumonia, UTIs, and STIs demonstrate largely comparable results across most measured indicators, with one statistically significant exception. In patients diagnosed with pneumonia (*n* = 21), the HaH group (*n* = 7) showed slightly higher rates of 3-day and 14-day ED revisits (14.3% and 28.6%, respectively) compared to the control group (7.1% and 7.1%), although these differences were not statistically significant (*p* = 0.60 and *p* = 0.19, respectively) (Supplementary Table S1). Similarly, readmission rates at 3 and 14 days, as well as 7-day and 30-day mortality, did not differ significantly between the two groups, indicating that HaH care may be a viable alternative to inpatient treatment for pneumonia without compromising short-term safety or leading to increased healthcare utilization. For UTI patients (*n* = 78), outcomes remained consistent across groups. Both HaH (*n* = 26) and in-hospital patients (*n* = 52) had identical 3-day ER revisit and readmission rates (7.7%), and although 14-day ED revisits and readmissions were slightly lower in the HaH group (19.2% vs. 34.6% and 23.1% vs. 30.8%, respectively), the differences were not statistically significant (*p* = 0.16 and *p* = 0.48). Mortality within 7 and 30 days was observed only in the HaH group (3.8% and 7.7%, respectively), but again, without reaching statistical significance (Supplementary Table S2).

The most notable finding emerged among STI patients (*n* = 81), where the 14-day ER revisit rate was significantly lower in the HaH group (7.4%) compared to the in-hospital group (27.8%), with an odds ratio of 0.21 (95% CI: 0.04–0.99, *p* = 0.04) (Supplementary Table S3). This suggests a potential advantage of HaH care in reducing post-discharge healthcare utilization among this specific patient population. Other outcome measures in this group, including readmission and mortality rates, also favored the HaH group numerically but did not achieve statistical significance. Importantly, no 7-day mortality was reported in either group for STI patients, reinforcing the safety of HaH for managing this type of infection. Further analysis compared the incidence of complications between patients in HaH and in-hospital control groups. Overall, the control group exhibited a higher rate of infections (14.2%) compared to the HaH group (10.0%). Cardiovascular and respiratory issues were slightly more common in the HaH group (3.3%) than in the control group (2.5%). Gastrointestinal problems (4.2% vs. 0.0%), neurological symptoms (1.7% vs. 0.0%), and other complications (4.2% vs. 3.3%) were more prevalent in the in-hospital group (Figure 2).

## Discussion

### Cost-effectiveness and system-level implications

This preliminary study represents the first evaluation of clinical outcomes associated with the HaH model in Taiwan., specifically focusing on older residents of nursing homes. Our findings offer compelling evidence that HaH can serve as a safe and feasible alternative to traditional hospitalization, particularly for managing common infections such as STIs, UTIs, and pneumonia. A key finding of this study was the significantly shorter duration of care episodes in the HaH group. On average, HaH patients received care for 6.6 days, compared to 11.8 days in the in-hospital group (*p* < 0.001) (Figure 1). This trend remained consistent across all major diagnostic categories: pneumonia (8.4 vs. 15.1 days, *p* = 0.005), urinary tract infections (6.3 vs. 10.3 days, *p* < 0.001), and skin and soft tissue infections (6.3 vs. 12.4 days, *p* < 0.001) (Table 2). Comparable results have been reported in several other studies, further supporting the time efficiency of the HaH model (13, 21–23). However, the average length of care episodes in our study was longer than that reported in some previous studies. This discrepancy may be attributed to our study population's older age and poorer baseline functional status, factors known to prolong recovery time and increase care needs (19, 24). Despite this, our findings align with prior research demonstrating the cost-efficiency of HaH model. Levine's randomized controlled trial in the United States demonstrated that home hospitalization for acute conditions significantly reduced healthcare costs, 30-day readmissions, and the use of diagnostic testing (13). Similarly, an Israeli study reported that HaH care for infections such as pneumonia, cellulitis, and UTIs achieved 30%–40% cost reductions compared to traditional hospitalization (*p* < 0.05) (21). Notably, the cost savings observed in Taiwan were even more pronounced: our HaH program achieved a 46.0% cost reduction for UTIs and a striking 65.1% reduction for STIs, compared to 24.4% and 38.5%, respectively, in the Israeli study. These differences may reflect variations in healthcare system design, pricing structures, and hospital overhead. Taiwan's single-payer system, standardized care protocols, and cost control mechanisms (25) likely enhance the efficiency of home-based care. Furthermore, the targeted implementation of Taiwan's HaH pilot, emphasizing early intervention and avoidance of unnecessary hospitalizations, may contribute to greater economic impact. These findings highlight the HaH model's potential to provide timely, effective acute care while significantly reducing costs.

### Clinical safety and comparative outcomes

Clinical outcomes between HaH and hospital-based care were generally comparable across key safety indicators, including 3-day and 14-day ED revisit rates, readmission rates, and short-term mortality. Among patients with pneumonia, the HaH group demonstrated numerically higher ED revisit rates at both 3 and 14 days (14.3% and 28.6% vs. 7.1% and 7.1%, respectively); however, these differences were not statistically significant. Similarly, in patients with UTIs, no significant differences were found in readmission or ED revisit rates, although slightly lower rates were observed in the HaH group at 14 days. These findings are consistent with previous research, which has shown that HaH can achieve similar or even better safety outcomes compared to conventional inpatient care. For instance, Levine et al. (2020) reported no significant differences in 30-day readmission or ED revisit rates between HaH and traditional hospitalization groups (13). Similarly, a systematic review involving data from 959 participants found that HaH care was associated with comparable or lower rates of readmissions and emergency department visits across a range of patient populations (26).

The most significant outcome difference emerged in the STI subgroup. The 14-day ER revisit rate was significantly lower in the HaH group (7.4%) compared to the in-hospital group (27.8%, *p* = 0.04), with an odds ratio of 0.21 (95% CI: 0.04–0.99). This suggests that HaH may offer particular benefits for patients with STIs in terms of reducing post-discharge healthcare utilization.

**Figure 3:**
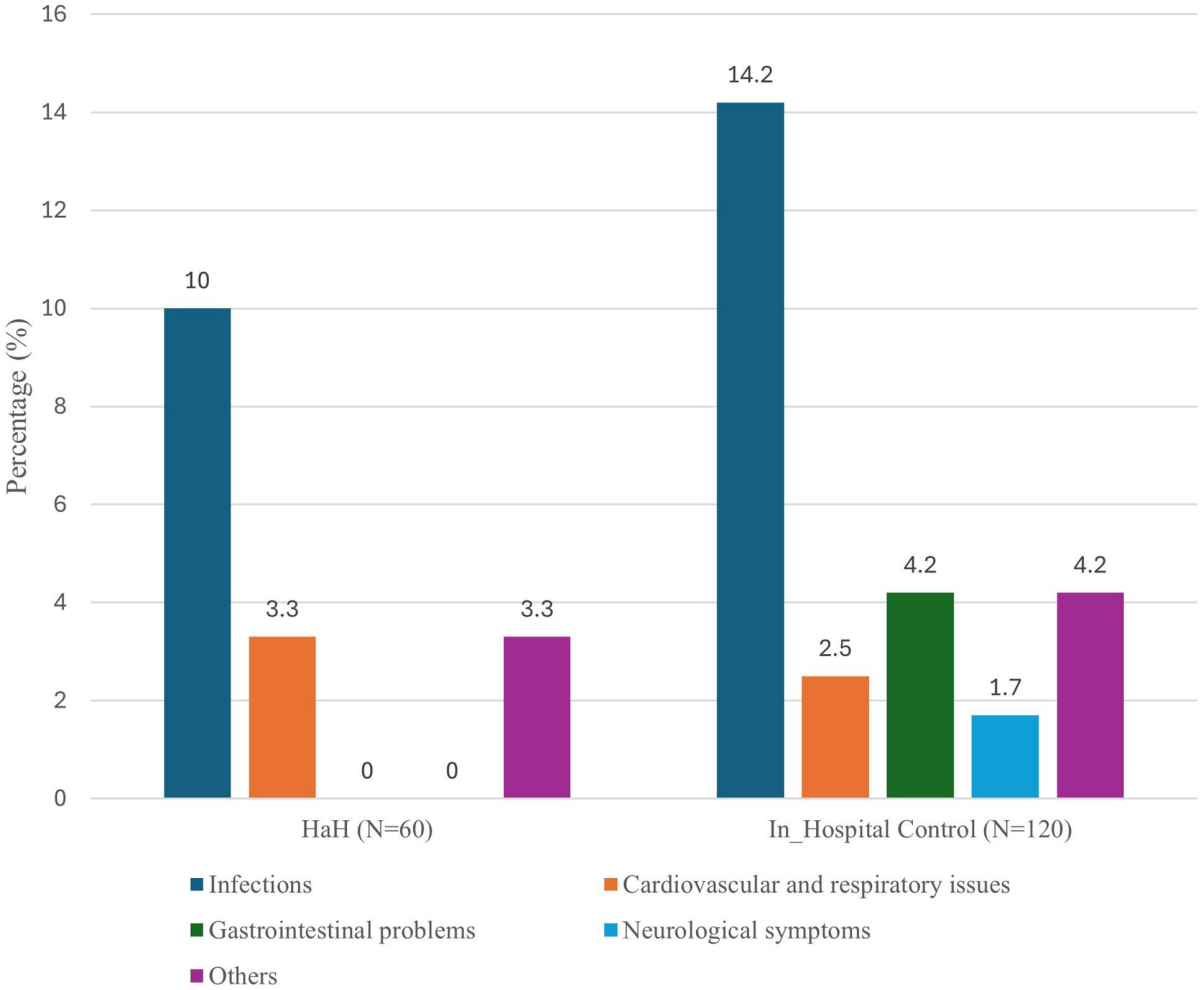
The incidence of complications in patients between HaH and in-hospital control groups.

Additionally, our study demonstrated that patients managed under the HaH model experienced fewer infections compared to those receiving in-hospital care (10.0% vs. 14.2%) (Figure 3). This finding is consistent with prior evidence from a comprehensive systematic review of 61 randomized controlled trials, which concluded that HaH care was associated with significantly lower risks of healthcare-associated infections, mortality, hospital readmissions, and overall healthcare costs when compared to conventional inpatient care (27, 28). We observed slightly higher rates of respiratory and cardiovascular complications in the HaH group compared to hospitalized patients (3.3% vs. 2.5%), which may reflect differences in patient selection or care delivery in the home setting. Conversely, serious gastrointestinal and neurological complications occurred only among inpatients (4.2% vs. 0%), suggesting that home care may mitigate risks related to hospital-acquired gastrointestinal issues or mild delirium. Our findings align with those from previous studies. For instance, a randomized trial by Caplan et al. (28) reported significantly fewer bowel complications and urinary complications in the HaH group compared to hospitalized patients. Similarly, a trial focusing on patients with dementia found that fewer individuals in the HaH group were prescribed antipsychotic medications at discharge (29), suggesting potential benefits of the home environment in reducing the use of potentially inappropriate medications. Collectively, these findings reinforce the growing literature that HaH care not only delivers medical outcomes on par with traditional hospitalization but may also reduce common complications, especially infectious and gastrointestinal in nature.

### Strength and weakness

The study included 180 participants, with 60 in the HaH group and 120 in the hospital based control group. Both groups were well matched in baseline characteristics, including age, sex, primary diagnosis, and functional status, enhancing the validity of outcome comparisons. A key strength of this study is its integrated care model. All healthcare providers, including physicians, nurses, and allied health professionals, were part of the same institution, Chiayi Hospital, Ministry of Health and Welfare, ensuring consistent protocols and effective coordination. This likely contributed to the safe and efficient delivery of HaH services and helped reduce care fragmentation, which is a common challenge in home based care.

However, the study has several limitations. The sample size was modest, particularly within diagnostic subgroups such as the 21 patients with pneumonia, which limits statistical power to detect meaningful differences. Matching was restricted to variables with complete and consistent documentation across both settings (age, sex, diagnosis, and admission timing). Comorbidities and detailed functional assessments were not used for matching due to variability in retrospective documentation. The follow up period was also short, limited to 30 days, which prevented the evaluation of long term outcomes such as functional recovery, quality of life, or caregiver burden. In addition, the study was conducted within a single healthcare institution, which may limit the generalizability of the findings to other settings with different infrastructure, care models, or patient populations. Additionally, although not directly addressed in this study, the issue of polypharmacy is especially relevant in institutionalized older adult populations. Previous research has shown a strong association between polypharmacy and increased risks of hospitalization, ED visits, and mortality (15, 17). Future HaH studies should explore whether home-based care can support better medication management and potentially reduce polypharmacy-related risks.

## Conclusion

This study offers the first empirical assessment of Taiwan's HaH model for managing acute infections in long-term care residents. The results indicate that HaH is a safe, practical, and cost-saving alternative to traditional hospitalization for conditions such as pneumonia, urinary tract infections, and soft tissue infections. HaH patients experienced shorter care episodes, significantly reduced costs, and comparable or better outcomes in ED revisits, readmissions, and complications. Because all HaH teams operate under standardized NHIA regulations, our findings offer relevant insights while acknowledging inherent single-site limitations.

A notable finding was the significantly lower 14-day ED revisit rate among patients with soft tissue infections, suggesting reduced post-discharge healthcare use. HaH care was also associated with fewer hospital-acquired complications, particularly gastrointestinal and neurological issues, supporting its safety and clinical viability.

These findings highlight HaH as a promising strategy to improve care efficiency and continuity for Taiwan's aging population. Future research should focus on larger, multi-site cohorts with extended follow-up to assess long-term outcomes, quality of life, caregiver burden, and medication management.

## Data Availability

The raw data supporting the conclusions of this article will be made available by the authors, without undue reservation.
