# Supplementary material for: Pioneering hospital-at-home in Taiwan: early clinical outcomes from the first cohort of nursing home older adults
Source: Front Health Serv. 2025 Dec 12;5:1696104. doi: 10.3389/frhs.2025.1696104 (PMC12741059; doi:10.3389/frhs.2025.1696104)
Supplement: Supplementary file 1 [file Table1.docx]

**Supplementary Table S1. Clinical Outcomes of patients with pneumonia between HaH and In Hospital Control Groups**

| **Clinical Outcome of Pneumonia Patients** | **HaH**  **(n=7)** | **In Hospital Control (n=14)** | **OR**  **95% CI** | ***p*-value** |
| --- | --- | --- | --- | --- |
| 3-day ED revisits rate, n (%) | 1 (14.3%) | 1 (7.1%) | 2.17  (0.12-40.81) | 0.60 |
| 14-day ED revisits rate, n (%) | 2 (28.6%) | 1 (7.1%) | 5.20  (0.38-70.90) | 0.19 |
| 3-day readmission rate, n (%) | 0 (0.0%) | 2 (14.3%) | 0.86  (0.69-1.06) | 0.29 |
| 14-day readmission rate, n (%) | 1 (14.3%) | 2 (14.3%) | 1.00  (0.08-13.4) | 1.00 |
| 7-day mortality, n (%) | 1 (14.3%) | 0 (0.0%) | 1.17  (0.86-1.58) | 0.15 |
| 30-day mortality, n (%) | 1 (14.3%) | 1 (7.1%) | 2.17  (0.12-40.81) | 0.60 |

**Supplementary Table S2. Clinical Outcomes of patients with UTIs between HaH and In Hospital Control Groups**

| **Clinical Outcome of UTIs Patients** | **HaH**  **(n=26)** | **In Hospital Control (n=52)** | **OR**  **95% CI** | ***p*-value** |
| --- | --- | --- | --- | --- |
| 3-day ED revisits rate, n (%) | 2 (7.7%) | 4 (7.7%) | 1.00  (0.17-5.85) | 1.00 |
| 14-day ED revisits rate, n (%) | 5 (19.2%) | 18 (34.6%) | 0.45  (0.15-1.39) | 0.16 |
| 3-day readmission rate, n (%) | 2 (7.7%) | 4 (7.7%) | 1.00  (0.17-5.85) | 1.00 |
| 14-day readmission rate, n (%) | 6 (23.1%) | 16 (30.8%) | 0.68  (0.23-2.00) | 0.48 |
| 7-day mortality, n (%) | 1 (3.8%) | 0 (0.0%) | 1.04  (0.96-1.12) | 0.16 |
| 30-day mortality, n (%) | 2 (7.7%) | 0 (0.0%) | 1.08  (0.97-1.21) | 0.11 |

**Supplementary Table S3. Clinical Outcomes of patients with STIs between HaH and In Hospital Control Groups**

| **Clinical Outcome of STIs Patients** | **HaH**  **(n=27)** | **In Hospital Control (n=54)** | **OR**  **95% CI** | ***p*-value** |
| --- | --- | --- | --- | --- |
| 3-day ED revisits rate, n (%) | 1 (3.7%) | 7 (13.0%) | 0.26  (0.03-2.22) | 0.19 |
| 14-day ED revisits rate, n (%) | 2 (7.4%) | 15 (27.8%) | 0.21  (0.04-0.99) | 0.04* |
| 3-day readmission rate, n (%) | 1 (3.7%) | 7 (13.0%) | 0.26  (0.03-2.22) | 0.19 |
| 14-day readmission rate, n (%) | 2 (7.4%) | 12 (22.2%) | 0.28  (0.06-1.36) | 0.10 |
| 7-day mortality, n (%) | 0 (0.0%) | 0 (0.0%) | NA | NA |
| 30-day mortality, n (%) | 1 (3.7%) | 3 (5.6%) | 0.65  (0.07-6.60) | 0.72 |

**p<0.05*
